# Intraoperative Low-Dose S-Ketamine Reduces Depressive Symptoms in Patients with Crohn’s Disease Undergoing Bowel Resection: A Randomized Controlled Trial

**DOI:** 10.3390/jcm12031152

**Published:** 2023-02-01

**Authors:** Zhen Zhang, Wen-Hao Zhang, Yin-Xiao Lu, Bo-Xuan Lu, Yi-Bo Wang, Li-Ying Cui, Hao Cheng, Zhen-Yu Yuan, Jie Zhang, Da-Peng Gao, Jian-Feng Gong, Qing Ji

**Affiliations:** 1 Department of Anesthesiology, Jinling Hospital, Medical School of Nanjing University, Nanjing 210016, China; 2 Department of General Surgery, Jinling Hospital, Medical School of Nanjing University, Nanjing 210016, China

**Keywords:** S-ketamine, postoperative depression, Crohn’s disease, postoperative pain

## Abstract

**Background:** Patients with Crohn’s disease (CD) undergoing bowel resection often suffer from depression and acute pain, which severely impairs their recovery. We aimed to investigate the effects of S-ketamine preconditioning on postoperative depression in patients with CD undergoing a bowel resection with mild to moderate depression and to observe whether it can relieve postoperative pain and anti-inflammation. **Methods:** A total of 124 adult patients were randomized into one of the two groups. Patients in the S-ketamine group received a 0.25 mg/kg S-ketamine intravenous drip under general anesthesia induction, followed by a continuous infusion of S-ketamine with 0.12 mg/kg/h for more than 30 min through target-controlled infusion. Patients in the placebo group received 0.9% saline at an identical volume and rate. The primary outcome measure was the 17-item Hamilton depression Scale (HAMD-17). The secondary outcomes were scores on the following questionnaires: a nine-item patient health questionnaire (PHQ-9); a quality of recovery (QoR-15) form; and a numeric rating scale (NRS). Additional secondary outcomes included the levels of C-reactive protein (CRP) and interleukin 6 (IL-6) on postoperative days (PODs) 1, 3, and 5, the length of hospital stay, and opioid use throughout the hospital stay. **Results:** The scores of PHQ-9 and HAMD-17 in the S-ketamine group were lower than those in the placebo group on postoperative days (PODs) 1, 2, and 7 (*p* < 0.05). The scores of QoR-15 in the S-ketamine group were higher than those in the placebo group on postoperative days (PODs) 3 and 5 (*p* < 0.05). The NRS scores of PACU, postoperative days 1 and 2 in the S-ketamine group were lower than those in the placebo group (*p* < 0.05). There was no significant difference in the CRP and IL-6 levels on postoperative days (PODs) 1, 3, and 5, postoperative complications, and hospital stay between the two groups (*p* > 0.05). **Conclusions:** The trial indicated that the intraoperative administration of low-dose S-ketamine could alleviate mild-to-moderate depressive symptoms and postoperative pain in patients with Crohn’s disease undergoing bowel resection without worsening their safety.

## 1. Introduction

Crohn’s disease (CD) is a chronic inflammatory disease of the gastrointestinal tract with symptoms evolving in a relapsing and remitting manner [1]. Typically, the peak incidence of CD is 18 to 35 years [2], which means that patients with CD will be plagued by the disease during their most precious years, and nearly 35% of them will develop depressive symptoms [3]. For 70% of patients with CD who need surgery [4], the incidence of preoperative depression may be higher. The CD cohort with a diagnosable psychological condition has been shown to experience a higher rate of disease exacerbation than the CD cohort without psychological complications [5,6]. At the same time, this depressive mood may make postoperative recovery more difficult, so it is necessary to alleviate postoperative depression.

Ketamine, a widely used anesthetic, is also used to treat depression [7]. The most-used ketamine in clinical practice is racemic ketamine, but its use is associated with many complications such as psychotic adverse effects and neurotoxicity [8,9]. In recent years, S-ketamine has received attention for its better efficacy and fewer complications [10,11]. In 2019, the Food and Drug Administration (FDA) approved S-ketamine nasal spray for the treatment of refractory depression and subsequently received approval from numerous health authorities around the world [12]. This proves that S-ketamine can provide a rapid antidepressant effect in patients with depression in a non-surgical setting. John et al. found that after blocking the NMDA (N-methyl-D-aspartate) receptor, S-ketamine can increase the number and function of synapses through a series of signal transductions, thus rapidly replenishing the reduced dendritic spines due to stress and depression in the circuits involved in mood regulation [13]. However, whether S-ketamine affects surgical patients is inconclusive, mainly because of the differences in the type of surgery, the dosage administered, the interaction with analgesics, and the evaluation tools implemented. Studies have shown that small doses of S-ketamine in breast cancer surgery and cervical cancer surgery can reduce postoperative depression [14,15,16]. However, the effects of S-ketamine on postoperative depression (POD) and pain in patients with CD have not been studied.

This study aimed to conduct a randomized, double-blind controlled trial to investigate the effects of S-ketamine preconditioning on postoperative depression in patients with CD undergoing bowel resection with mild-to-moderate depression and to observe whether it can relieve postoperative pain and anti-inflammation. We hypothesized that the continuous intraoperative infusion of low-dose S-ketamine could effectively reduce postoperative depression in patients with CD undergoing bowel resection with mild-to-moderate depression.

## 2. Methods

### 2.1. Study Design

In this double-blind, randomized prospective trial, we investigated whether the intraoperative use of low-dose S-ketamine could reduce postoperative pain scores and improve anxiety and depression in patients with CD. The research protocol was approved by Jinling Hospital affiliated with Nanjing University Medical College in Nanjing City, Jiangsu Province, and the written informed consent of all subjects was obtained. The study was registered with the ClinicalTrials.gov, and the registration number is NCT05506787.

Patients with CD who were hospitalized and required surgery from 1 September 2020 to 1 March 2022 were included. The inclusion criteria were as follows: (a) subjects who volunteered and signed informed consent for the trial, (b) patients aged between 18 and 60, (c) patients with CD who had undergone surgery, (d) American Society of Anesthesiologists (ASA) grade I~III, and (e) a Hamilton depression score of 8–24. Exclusion criteria included: (a) allergy to the narcotic drugs or ketamine used, (b) patients with other serious systemic diseases (including severe heart, kidney, and liver disease), (c) chronic opioid therapy (more than twice a week for more than three months), (d) inability to understand the digital pain scale, and (e) patients who had received antidepressant treatment within the last month.

### 2.2. Randomization and Masking

Randomization assignments were conducted from a computer-generated random number table. Group distributions and research drug regimens were hidden in sequentially numbered, sealed, opaque envelopes. On the day of the operation, an assistant who had no knowledge of the research program opened the envelope in the order in which the subject registered, reloaded the research drug into a 50 mL syringe, and then labeled the syringe with the designated subject ID. The subjects, anesthesiologists, and follow-up investigators were masked from the subjects’ group assignments. This information was concealed until the recruitment and follow-up of the subjects were completed.

### 2.3. Procedures

In this randomized, double-blind, controlled trial, 124 patients who met the inclusion criteria were randomly assigned to two groups using prepared sealed envelopes and received either an S-ketamine grouping or a placebo grouping. The patients in the S-ketamine group received a 0.25 mg/kg intravenous S-ketamine (Jiangsu Hengrui Pharmaceutical Co., Ltd., Lianyungang, Jiangsu, China) drip under a general anesthesia induction, followed by a continuous infusion of S-ketamine of 0.12 mg/kg/h for more than 30 min through target-controlled infusion. Drug A was S-ketamine diluted to 1 mg/mL with normal saline, total 50 mL). Patients in the placebo group received an intravenous infusion of 0.9% saline during anesthesia induction, and this was maintained throughout the infusion pump of drug B (50 mL 0.9% saline) for more than 30 min. Patients normally fasted for 12 h. After a patient arrived in the operating room, a peripheral venous access was established in the right or left antecubital vein, and 0.9% normal saline was injected at a rate of 20 mL/min, and blood pressure, blood oxygen saturation, end-tidal CO_2_ pressure, and electrocardiogram monitoring were measured at the same time using a Dash-4000 monitoring instrument. Induction of anesthesia was conducted with midazolam 40 μg/kg, either A or B drug 0.25 mg/kg (20 s post-injection), sufentanil 3 μg/kg, propofol 1–2 mg/kg, and cisatracurium 0.2 mg/kg. Continuous infusion of drug A or B at a rate of 0.12 mg/kg/h was conducted through an infusion pump. Anesthesia was maintained using a target-controlled infusion of propofol and remifentanil. Then, remifentanil 0.2–0.5 mg/(kg·min), dexmedetomidine 0.5 μg/(kg·h), cisatracurium 5–8 mg/(kg·min), and 2–3% sevoflurane inhalation anesthesia was intravenously injected. All analgesia was performed by the same team, and the anesthesiologist was blinded to the drugs administered after analgesia induction. Analgesia and surgical procedures were performed by the same team following the same treatment protocol. After surgery, all patients received patient-controlled intravenous analgesia (PCIA) containing 5 mg of dezocine injection (4 vials), 50 μg of sufentanil citrate (1 vial), and 8 mg of ondansetron (1 vial) for continuous PCIA for 24 h.

### 2.4. Outcome Measures

The primary outcome measured was the 17-item Hamilton depression scale 17-item (HAMD-17). The HAMD-17 is a 17-item tool that determines the severity of depressive symptoms. Higher scores indicate a greater severity of depression. The secondary outcomes were scores on the following questionnaires: first, the nine-item patient health questionnaire (PHQ-9) was used. This nine-item questionnaire is an efficient tool for evaluating depression severity and consists of the nine criteria upon which the diagnosis of depressive disorders is based. Higher scores represent a more severe condition. second, a quality of recovery (QoR-15) form was used. The QoR-15 comprised 15 questions (maximum score, 150; good recovery, 118). Each question used a 10-point scale ranging from 0 = “none of the time” to 10 = “all of the time” (scoring was reversed for negative questions). Third, a numeric rating scale (NRS) was used. The scores ranged from 0–10 points, with higher scores indicating a greater pain intensity. HAMD-17 and PHQ-9 are well-established questionnaires for the assessment of mood and are often used in depression studies. The QoR-15 assessed the overall satisfaction with postoperative recovery. The NRS assessed pain. HAMD-17, PHQ-9, and QoR-15 were assessed prior to surgery in the preadmission clinic. Prior to discharge from the PACU, the NRS was assessed on postoperative days (PODs) 1, 2, and 7. The questionnaires (HAMD-17, PHQ-9, and QoR-15) were assessed on postoperative days (PODs) 1, 3, 7, and 30. If a patient had been discharged prior to POD 3 or 7, a member of the study team contacted the patient by telephone to complete the questionnaires. Additional secondary outcomes included the levels of C-reactive protein (CRP) and interleukin 6 (IL-6) on postoperative days (PODs) 1, 3, and 5, the length of hospital stay, and opioid use throughout the hospital stay. All opioid doses were converted to a morphine intravenous equivalent (with www.hopweb.org (accessed on 4 April 2022)). Exploratory secondary measurements included the measurement of serum markers. However, all patients refused additional blood analysis. All patients were followed up for at least three months.

### 2.5. Measurement of Biomarkers

The serum concentration of IL-6 was tested by a solid-phase enymze-linked immunosorbent assay with a capture and peroxidase-labeled tracer antibody (Bio Legend, San Diego, CA, USA). The oxidase converted phenylenediamine-dihydrochloride into a yellow color, which was proportional to the concentration of IL-6. The CRP concentration was part of the routine perioperative laboratory tests and was retrieved from the patients’ computerized medical charts.

### 2.6. Statistical Analysis

The level of hypothesis testing was determined, the value of α = 0.05 was taken, and the test efficiency was 90%. According to the pre-test and the other existing literature results, σ = 10, the effect difference D1 = 5, and the dropout rate was calculated as 5%. The sample size calculation software PASS15.0 was used. A total of 60 patients per arm were enrolled to allow for potential dropouts.

The continuous results were analyzed by the Kolmogorov–Smirnov test to judge the normality of its distribution. The measured data according to the normal distribution were expressed as the average ± standard deviation, and the measured data of the non-normal distribution were expressed as the median (quartile spacing). The independent t-test, the Mann–Whitney U test, the χ^2^ test, and the Fisher exact test were used to compare the baseline characteristics of each group as appropriate.

The fixed effects of the intervention (group: S-ketamine vs. placebo), time, and group-by-time interactions on the different end points (postoperative HAMD-17, PHQ-9, QoR-15, NRS, CRP, and IL-6) were modelled using the linear mixed-effect model with random intercepts for the subjects. Akaike’s information criteria were used to select the covariance structure for the residuals. All available data were used. In the presence of a group-by-time interaction, group comparisons at individual time points were conducted (contrast analysis), and the results were adjusted for multiple comparisons using the Bonferroni correction. A two-sided *p* value of less than 0.05 was considered significant. Statistical analysis was conducted using the SPSS 25.0 software (IBM, Armonk, NY, USA).

## 3. Result

**Figure 1:**
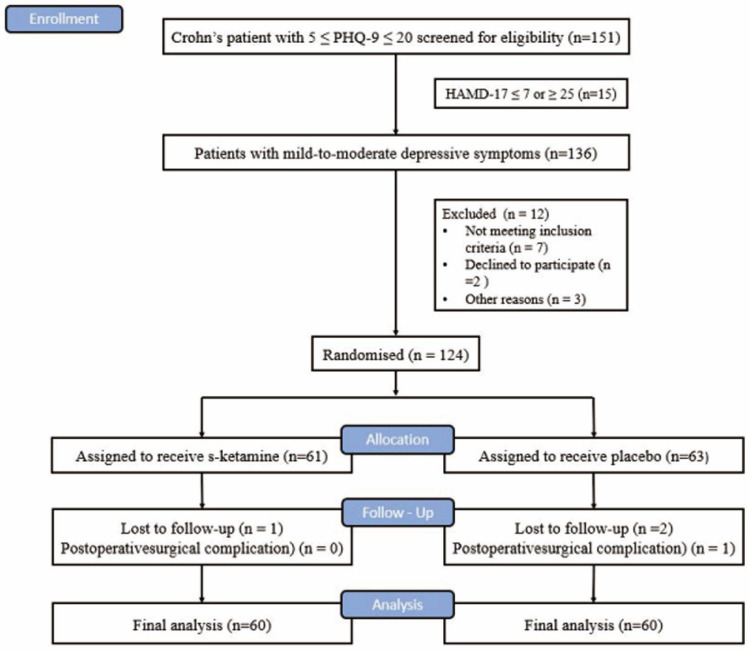
CONSORT flow chart.

From 1 September 2020 to 1 March 2022, 151 patients with CD with intestinal obstruction with a PHQ-9 score of 5–20 were screened in Jinling Hospital affiliated with the Medical College of Nanjing University (Figure 1). Finally, 124 patients were enrolled and randomly assigned to either the S-ketamine group (*n* = 61) or the placebo group (*n* = 63). One case lost follow-up in the S-ketamine group and three cases lost follow-up in the placebo group. Finally, data from the 60 subjects in the S-ketamine group and the placebo group were included in the analysis (Figure 1). There were no differences in the demographics, baseline assessment, and general intraoperative parameters (Table 1) between the two groups.

**Figure 2:**
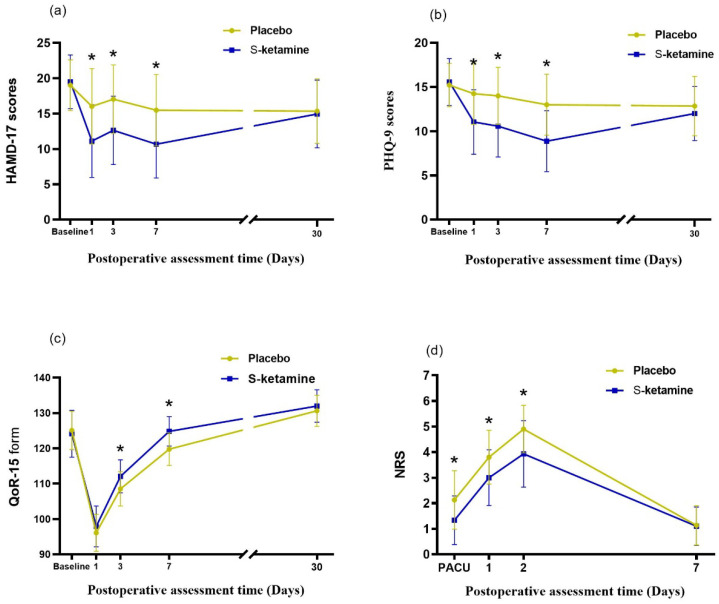
Trends in depressive scores after surgery. (**a**) HAMD-17 scores of enrolled patients; (**b**) PHQ-9 scores of enrolled patients. The line plot exhibits the mean scores for each group, and the error bars represent the SD for timepoints. The HAMD-17 and PHQ-9 scores of the S-ketamine group were significantly lower than those of the placebo group on the 1st, 3rd, and 7th day post operation (Bonferroni’s adjusted *p* < 0.001). (**c**) QoR-15 scores at 1 day, 3 days, 7 days, and 30 days after operation; the QoR-15 scores at 3 days and 7 days after operation in the S-ketamine group were significantly higher than those of the placebo group (Bonferroni’s adjusted *p* < 0.001). (**d**) NRS scores at PACU 1 day, 2 days, and 7 days after operation; the NRS scores of PACU 1 day and 2 days after operation in the S-ketamine group decreased significantly (Bonferroni’s adjusted *p* < 0.001). The symbol * represents *p* < 0.001. HAMD-17, seventeen-item Hamilton depression scale; SD, standard deviation; PHQ-9, nine-item patient health questionnaire. QoR-15, quality of recovery form. NRS, numeric rating scale; PACU, post-anesthesia care unit.

The linear mixed model was used to examine the interactions between group and time at different timepoints before and after surgery. The overall change in the HAMD-17 score over time is shown in Figure 2a. There was a statistically significant difference in the interaction between the intervention measures of the two groups and time (*F* = 84.974, *p* < 0.001). There was a significant difference in the magnitude of the change on day 1 (*F* = 32.846, *p* < 0.001), day 3 (*F* = 26.686, *p* < 0.001), and day 7 (*F* = 31.738, *p* < 0.001) after operation, but no significant differences between the groups were detected at day 30 (*F* = 0.219, *p* = 0.641), indicating that the intervention measures were effective in decreasing the postoperative HAMD-17 score within one week after considering the interactions between time and group. The total scores of the HAMD-17 (−5.3-point mean difference [SE 0.50] between arms; *p* < 0.001 [95% CI −6.3 to −4.3]) and PHQ-9 (−4.5-point mean difference [SE 0.38] between arms; *p* < 0.001 [95% CI −5.2 to −3.7]) scales improved from the baseline to day 7 in the S-ketamine group (Table 2).

The change in thePHQ-9 score over time is shown in Figure 2b. There was a statistically significant difference in the interaction between the intervention measures of the two groups and time (*F* = 86.994, *p* < 0.001). There was a significant difference in the magnitude of change on day 1 (*F* = 29.425, *p* < 0.001), day 3 (*F* = 33.872, *p* < 0.001), and day 7 (*F* = 48.697, *p* < 0.001) after operation, but no significant differences between the groups were detected at day 30 (*F* = 2.076, *p* = 0.152), indicating that the intervention measures were effective in decreasing the postoperative PHQ-9 score within one week after considering the interactions between time and group.

The change in the QoR-15 score over time is shown in Figure 2c. There was a statistically significant difference in the interaction between the intervention measures of the two groups and time (*F* = 33.395, *p* < 0.001). There was a significant difference in the magnitude of the change on day 3 (*F* = 14.141, *p* < 0.001) and day 7 (*F* = 28.427, *p* < 0.001) after operation, but no significant differences between the groups were detected on day 1 (*F* = 3.703, *p* = 0.056) and day 30 (*F* = 2.045, *p* = 0.155), indicating that the intervention measures were effective in decreasing the postoperative QoR-15 score after considering the interactions between time and group. There was no significant difference in the length of stay between the treatment and placebo groups (*p* = 0.185; Table 3).

The change in the NRS score over time is shown in Figure 2d. There was a statistically significant difference in the interaction between the intervention measures of the two groups and time (*F* = 8.348, *p* < 0.001). There was a significant difference in the magnitude of the change at PACU (*F* = 20.428, *p* < 0.001), day 1 (*F* = 22.130, *p* < 0.001), and day 2 (*F* = 24.810, *p* < 0.001) after operation, but no significant differences between the groups were detected at day 7 (*F* = 0.034, *p* = 0.854), indicating that the intervention measures were effective in decreasing the postoperative NRS score within two days after considering the interactions between time and group. The consumption of postoperative opioids used in the S-ketamine group was significantly lower than that in the placebo group within 2 days after surgery ((16.4 [standard deviation {SD} = 5.9] vs. 23.9 [SD = 8.4]; *p* < 0.05; Table 3).

**Figure 3:**
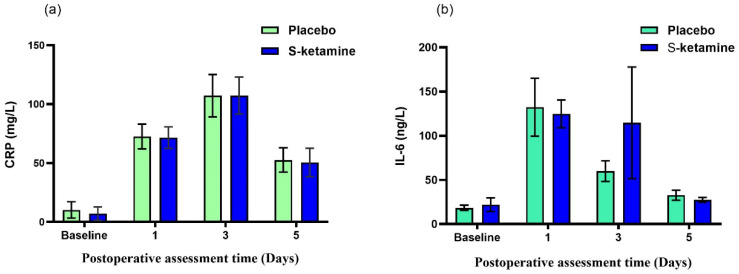
The change in CRP and IL-6 over time after surgery. (**a**) The values decreased over time (time effect *F* = 169.501, *p* < 0.001), but no significant differences between groups were seen (group effect *F* = 0.058, *p* = 0.811; group-by-time interaction *F* = 0.048, *p* = 0.986). (**b**) The values decreased over time (time effect *F* = 8.230, *p* < 0.001), but no significant differences between groups were seen (group effect *F* = 0.282, *p* = 0.597; group-by-time interaction *F* = 0.686, *p* = 0.561). CRP, C-reactive protein; IL-6, interleukin 6.

The change in the RCP value over time is shown in Figure 3a. The values decreased over time (time effect *F* = 169.501, *p* < 0.001), but no significant differences between the groups were seen (group effect *F* = 0.058, *p* = 0.811; group-by-time interaction *F* = 0.048, *p* = 0.986). The change in the IL-6 value over time is shown in Figure 3b. The values decreased over time (time effect *F* = 8.230, *p* < 0.001), but no significant differences between the groups were seen (group effect *F* = 0.282, *p* = 0.597; group-by-time interaction *F* = 0.686, *p* = 0.561).

There was no significant difference in the side effects between the treatment and placebo groups (Table 3). The most common side effects in both groups were dizziness, nausea, and irritability. All side effects were self-limited and did not require medical treatment except for verbal reassurance.

## 4. Discussion

In this randomized controlled trial involving patients with CD undergoing bowel resection with mild-to-moderate depressive symptoms, the intraoperative administration of low-dose S-ketamine could reduce the depressive symptoms and improve the quality of recovery for 1 week and relieve pain for two days. However, we did not find that low-doses of S-ketamine had an anti-inflammatory effect in these patients. In addition, S-ketamine did not increase the risk of psychiatric side effects or postoperative complications.

In many studies, S-ketamine has been shown to have rapid antidepressant effects. Our findings increased the possibility that the intraoperative use of S-ketamine may improve postoperative mood and enhance resilience in a stressful surgical environment. In 2019, Liu et al. reported that in breast cancer patients with preoperative depression, the intraoperative use of S-ketamine (0.125 mg/kg during induction and then given according to the experience of the anesthesiologist) could reduce the patient’s HAMD-17 score one month after surgery [14]. Wang et al. found that in mild-to-moderate depressive patients who underwent laparoscopic total hysterectomy for cervical cancer, the HAMD-17 score of the S-ketamine group (0.25 mg/kg/h after induction for 1 h) was significantly lower than that of the control group in the first 3 days post operation [15]. In this study, we found that the continuous intraoperative infusion of low-dose S-ketamine (0.25 mg/kg during induction, followed by 0.125 mg/kg/h) could effectively reduce the HAMD-17 score one week postoperatively in patients with CD with mild-to-moderate depression. The consistent results of these three studies indicated that the intraoperative administration of S-ketamine could reduce the postoperative HAMD-17 score of patients with mild-to-moderate depression, but the difference is found in the duration of the antidepressant effects. This may be related to the type of disease, dosage, and interaction with other drugs. We think that this difference may be related to the dose, although it was impossible to compare the doses of the three studies. Wang et al. found that the antidepressant effect of the high-dose group (0.5 mg/kg/h after 1 h of induction) was longer than that of the low-dose group (0.25 mg/kg/h after 1 h of induction) [15].

Patients with mild-to-moderate depressive symptoms screened with PHQ-9 and HAMD-17 before surgery participated in this study, and population homogeneity must be ensured to explore the antidepressant effects of S-ketamine. In this study, the patients responded well to most of the projects in terms of the PHQ-9 and HAMD-17 scores. Although the change in the total score is an essential indicator of the change in the severity of depressive symptoms, understanding the contribution of specific items to the change in total score is helpful in explaining the effects of the treatment. There were statistically significant improvements in items 1, 2, 4, and 6 of the PHQ-9 scale and items 1, 2, 7, and 10 of the HAMD-17 scale. Items 1 and 2 of PHQ-9 (“lack of interest/pleasure in doing things” and “feeling depressed, depressed or desperate”) were considered to be the main symptoms of depression; depression was diagnosed only when the patients accepted at least one of these two items. These results provided a way for patients to experience clinical improvement, which was more specific than the total score.

The Immune inflammatory reactions occur both in depressed patients and patients with inflammatory bowel disease (IBD), and some inflammatory factors are increased in the body as a result [17]. Abautret-Daly et al. [18] found that the level of serum inflammatory factors in IBD patients with higher anxiety and depression scores was higher than that in patients with lower anxiety and depression scores. Serum CRP levels have been used as markers of disease activity and remission in IBD. CRP increases the permeability of the blood–brain barrier, suggesting that the increase in serum CRP in IBD patients can lead to central nervous system damage [19]. Higher CRP levels suggest a higher risk of developing a new type of depression [20]. Ketamine has anti-inflammatory effects on depression [21]. Studies including patients with treatment-resistant depression showed that pro-inflammatory cytokines (mainly IL-6) decreased 4 h after a single intravenous injection of ketamine [22]. Another study showed that IL-6 and tumor necrosis factor α (TNF-α) levels decreased rapidly, and there was a correlation between the decline in TNF-α levels and the decrease in the Montgomery Asperger Depression rating scale (MADRS) scores [23]. In this study, we compared the CRP and IL-6 levels between the two groups at different time points postoperatively and found that the results were not statistically significant. This was different from previous studies [24], but it does not mean that S-ketamine does not have anti-inflammatory effects. Due to the large surgical trauma, the inflammatory response caused by surgery may far exceed the anti-inflammatory effects of ketamine. Therefore, further research is needed.

Many clinical trials have explored the use of ketamine to treat and prevent postoperative pain [25]. Low-dose ketamine (0.1–0.5 mg/kg) is thought to relieve postoperative pain management [26,27] when used as an adjuvant for local anesthetics, opioids, or other analgesics. In this study, we observed that the dosage of opioid analgesics in the S-ketamine group was significantly lower than that in the placebo group within 2 days post operation. This finding was similar to previous studies [28,29] but differed from the results of Wang et al.’s trials on the effects of a single subanesthetic dose of ketamine on pain and mood after laparoscopic surgery, which may be related to the type of medication, the mode of administration, and the nature of the disease. Of course, pain relief may help to improve depression. However, we found that there was no difference in the pain score between the two groups one week post operation, but there was a significant difference in the depression score, indicating that the postoperative analgesic effect of S-ketamine was not the main factor of the antidepressant. Recent evidence has demonstrated that opioid use is associated with severe infections and increased mortality among IBD patients [30,31,32]. At the same time, the reduction in the postoperative opioid dose is of great benefit to the recovery of intestinal function in patients with CD [33].

In our study, we observed that the incidence of hallucination and dizziness was lower in the S-ketamine group. The dose of ketamine was selected as the intervention dose based on the consensus in the literature, which was much lower than the anesthetic dose (2000–3000 ng mL^−1^) and did not cause strong hemodynamic fluctuations and severe psychiatric symptoms [34].

This study had several limitations. First, our study did not detect serum brain-derived neurotrophic (BDNF) and 5-hydroxytryptamine (5-HT) levels and did not assess the correlation between the HAMD-17 score and BDNF and 5-HT. Lepack et al. suggested that the release of BDNF is essential for the antidepressant behavior of ketamine [35]. The study also found that BDNF gene polymorphism is associated with the antidepressant efficacy of ketamine in patients with depression [36]. Second, the drugs such as Dexmedetomidine used during the surgery in this study may have interacted with the S-ketamine. Therefore, further research is needed to confirm these findings. Third, we only compared one dose of S-ketamine with a placebo but not with oral antidepressants, which often take more than 1 week to be effective. Finally, we only visited the patients 3 months post operation, which is far from enough to observe the disease development of patients with CD postoperatively.

## 5. Conclusions

In summary, this study suggested that among patients with CD undergoing bowel resection with mild-to-moderate depressive symptoms, the intraoperative administration of low-dose S-ketamine could reduce the patients’ depressive symptoms and improve the quality of recovery for 1 week and relieve pain for two days. Further multicenter studies are needed to investigate the long-term outcomes of S-ketamine in different surgical settings.

## Figures and Tables

**Table 1:** Baseline characteristics.

Variables	Placebo, *n* = 60	S-Ketamine, *n* = 60	*p*-Value
Age, *y*	35.2 ± 10.9	35.7 ± 9.3	0.774
Sex, male	45 (70%)	40 (66.7%)	0.315
Weight, kg	52.6 ± 8.4	51.0 ± 8.0	0.270
Height, cm	169.8 ± 8.4	168.3 ± 8.7	0.361
Body mass index, kg/m^2^	18.2 ± 2.3	18.0 ± 2.3	0.566
Education, senior middle school or above	57 (95%)	55 (91.7%)	0.359
Marriage status, married	38 (63.3%)	41 (68.3%)	0.564
Smoking history	10 (16.7%)	6 (10%)	0.283
Hypertension	1 (1.7%)	1 (1.7%)	1.000
Heart disorders	1 (1.7%)	0 (0)	0.500
Disease course, *y*	7.1 ± 4.9	8.2 ± 5.0	0.226
ASA (I/II)	13/47	7/53	0.315
PHQ-9	15.2 ± 2.45	15.6 ± 2.65	0.454
HAMD-17 score	19.5 ± 3.73	19.0 ± 3.59	0.475

Variables are described as number (%) and median (interquartile range). Abbreviations: ASA, American Society of Anesthesiologists; HAMD-17, seventeen-item Hamilton depression scale; PHQ-9, nine-item patient health questionnaire.

**Table 2:** HAMD-17 and PHQ-9 total score and change from baseline score at day 7.

Time Point	Absolute Score		Change from Baseline		Treatment Difference		
	Placebo	S-Ketamine	Placebo	S-Ketamine			
	Mean (SD)	Mean (SD)	Mean (SD)	Mean (SD)	Mean (SE)	95%CI	*p*-Value
**HAMD-17**							
Baseline	19.5 (3.73)	19.0 (3.59)					
Day 7	15.5 (5.04)	10.7 (4.80)	−3.5 (2.17)	−8.8 (3.27)	−5.3 (0.50)	−6.3 to −4.3	<0.001
**PHQ-9**							
Baseline	15.2 (2.45)	15.6 (2.65)					
Day 7	13.0 (3.44)	8.9 (3.45)	−2.2 (1.49)	−6.7 (2.53)	−4.5 (0.38)	−5.2 to −3.7	<0.001

HAMD-17, seventeen-item Hamilton depression scale; SD, standard deviation; PHQ-9, nine-item patient health questionnaire; SE, standard error.

**Table 3:** Intraoperative and postoperative data.

Variables	Placebo, *n* = 60	S-Ketamine, *n* = 60	*p*-Value
Laparoscopic surgery	18 (30%)	12 (20%)	0.206
Blood loss, mL	152.5 ± 89.5	174.2 ± 91.8	0.193
Anesthesia duration, min	140.0 (125.0–177.5)	150.0 (121.3–180.0)	0.721
Sufentanil, μg	31.8 (30.5–33.1)	30.4 (29.2.0–31.6)	0.110
Remifentanil, μg	623.5 (569.6–677.4)	610.3 (552.7–667.9)	0.739
Propofol, mg	945.9 (870.3–1021.5)	941.0 (853.5–1028.5)	0.993
Postoperative opioid used, mg	23.9 ± 8.4	16.4 ± 5.9	<0.050
Time to extubation, min	31.0 (20.5–58.0)	39.5 (22.0–50.8)	0.854
PPOI	7 (11.7%)	10 (16.7%)	0.432
Complication, *n* (%)			
Nausea	6 (10%)	3 (5%)	0.245
Dizzy	3 (5%)	5 (8.3%)	0.359
Dysphoria	1 (1.7%)	4 (6.7)	0.182
Length of stay, days	21 (17–25)	20 (16–22)	0.185

Variables are described as number (%) and median (interquartile range). Abbreviations: PPOI, prolonged postoperative ileus.

## Data Availability

The data generated in this study can be shared after a reasonable request to the corresponding author.
